# Growth faltering regardless of chronic diarrhea is associated with mucosal immune dysfunction and microbial dysbiosis in the gut lumen

**DOI:** 10.1038/s41385-021-00418-2

**Published:** 2021-06-22

**Authors:** Nicholas S. Rhoades, Sara M. Hendrickson, Kamm Prongay, Andrew Haertel, Leanne Gill, Robert A. Edwards, Laura Garzel, Mark K. Slifka, Ilhem Messaoudi

**Affiliations:** 1 Department of Molecular Biology and Biochemistry, University of California Irvine, Irvine, CA USA; 2 Division of Neuroscience, Oregon National Primate Research Center, Portland, OR USA; 3 Division of Comparative Medicine, Oregon National Primate Research Center, Oregon Health and Science University West Campus, Portland, OR USA; 4 California National Primate Research Center, Davis, Davis, CA USA; 5 Department of Pathology and Laboratory Medicine, University of California, Irvine, CA USA

## Abstract

Despite the impact of childhood diarrhea on morbidity and mortality, our understanding of its sequelae has been significantly hampered by the lack of studies that examine samples across the entire intestinal tract. Infant rhesus macaques are naturally susceptible to human enteric pathogens and recapitulate the hallmarks of diarrheal disease such as intestinal inflammation and growth faltering. Here, we examined intestinal biopsies, lamina propria leukocytes, luminal contents, and fecal samples from healthy infants and those experiencing growth faltering with distant acute or chronic active diarrhea. We show that growth faltering in the presence or absence of active diarrhea is associated with a heightened systemic and mucosal pro-inflammatory state centered in the colon. Moreover, polyclonal stimulation of colonic lamina propria leukocytes resulted in a dampened cytokine response, indicative of immune exhaustion. We also detected a functional and taxonomic shift in the luminal microbiome across multiple gut sites including the migration of *Streptococcus* and *Prevotella* species between the small and large intestine, suggesting a decompartmentalization of gut microbial communities. Our studies provide valuable insight into the outcomes of diarrheal diseases and growth faltering not attainable in humans and lays the groundwork to test interventions in a controlled and reproducible setting.

## Introduction

Despite significant improvements in healthcare delivery and sanitation, over 500,000 children under the age of 5 die of diarrheal disease every year^1^. In addition to high mortality rates, diarrhea early in life can have a lasting effect on host physiology^2^. Specifically, diarrhea results in a dangerous feedback loop of malnutrition^3–6^ and intestinal damage^3,7,8^, which ultimately leads to growth faltering, poor responses to oral vaccination^9–12^, and diminished resilience to subsequent enteric infections^13^. This cycle can occur even in the absence of overt diarrhea^14,15^. Indeed, growth faltering children in developing countries have been shown to have a significant enteric pathogen burden without acute disease^14,16,17^. These subclinical infections are believed to perpetuate intestinal damage and restrict healthy growth^18^. Moreover, unknown and emerging etiological agents are thought to play an underappreciated role in childhood diarrheal disease and growth faltering. Specifically, multiple large-scale international studies have found that less than 50% of diarrheal cases can be attributed to a specific pathogen^19,20^. This pattern is likely a combination of high pathogen burden in the absence of symptoms^19–21^ and the use of clinical tests that limit detection of novel pathogens not routinely detected^22,23^. More recent studies have begun to identify a set of non-traditional pathogens, especially in the genus *Campylobacter*, that are missed by clinical tests but highly abundant in developing countries^24–26^. Additional studies are needed to determine the role of these newly identified pathogens in the cycle of diarrheal disease, malnutrition, and growth faltering.

In addition, few studies have investigated diarrheal disease-induced damage within the gut itself. One study reported increased gut permeability and inflammation markers in duodenal biopsies obtained from Gambian infants with growth faltering compared to those obtained from healthy children living in the United Kingdom^9^. While this study reported an association between growth faltering and damage to the upper small intestine, the involvement of other gut sites remains unknown. More importantly, the interpretation of these observations is complicated by the lack of comparison to samples obtained from asymptomatic children living under the same conditions. Transcriptional analysis of fecal samples obtained from infants with environmental enteric dysfunction revealed increased expression of immune genes^27^; however, the cellular source of these expression changes remains unclear.

Alterations in mucosal immune cell frequency and function across the GI tract due to growth faltering/diarrheal disease have yet to be identified due to the challenges associated with the collection of intestinal biopsies from healthy infants and those experiencing growth faltering while controlling for key variables such as geographic location, diet, and socioeconomic status. A deeper understanding of the pleiotropic impact of diarrhea on GI immunological and microbial community composition is key to successfully identify sub-clinical cases and developing targeted treatments against growth faltering and malnutrition.

Malnutrition, growth faltering, and diarrhea have all been associated with dysbiosis of the gut microbiome at a taxonomic level using fecal samples^28–30^, as well as the persistence of an immature gut microbiome state^31,32^. Moreover, growth faltering is associated with the decompartmentalization of microbial communities as illustrated by the recent report of potential infiltration of nasopharyngeal microbes into the GI tract^33^. Interestingly, levels of enteric pathogens such as *Escherichia coli* and *Campylobacter spp*. are elevated in the fecal microbiome of children with malnutrition and growth faltering regardless of diarrhea^16,25,34,35^. However, the vast majority of clinical findings associated with diarrheal disease and growth faltering are centered around the small intestine, while the majority of microbiome data is generated from fecal samples. This dichotomy may conceal clinically relevant information and no studies to date have interrogated microbial changes within the multiple intestinal tract sections at the taxonomic or functional level.

Answering these questions would be greatly facilitated by the availability of an animal model that was susceptible to human enteric pathogens and displayed the hallmarks of growth faltering. Rhesus macaques are the gold standard model for the study of several infectious diseases due to their genetic and physiological similarity to humans. Rhesus macaques housed at the Oregon National Primate Research Center (ONPRC) and California National Primate Research Center (CNPRC) naturally develop the diarrheal disease at a predictable yearly rate with *Campylobacter* and *Shigella* being the primary pathogens isolated at clinic admission^36–38^. In addition, infant macaques that experience diarrhea suffer growth faltering^37^. Rhesus macaques also display a high pathogen burden even in the absence of diarrheal disease, as described in infants^19^. Moreover, we recently reported that the gut microbiome of infant rhesus macaques closely resembles that of children in the developing world where diarrheal disease and growth faltering is most prevalent^29^.

In this study, we leveraged the infant macaque model to characterize the impact of growth faltering with distant or active chronic diarrhea on the mucosal immune system and microbial communities throughout the intestinal tract. Our data revealed immunological and microbial dysregulation that was more pronounced in the colon. Specifically, we detected alterations in the frequency of immune cell populations and their response to polyclonal stimulation, as well as shifts in microbial communities along the entire GI tract, as well as the migration of microbes between gut sites suggestive of decompartmentalization. Shotgun metagenomics of luminal contents revealed key differences in the functional potential of luminal communities and led to the assembly of novel *Campylobacter* strains in animals with growth faltering. Together, our findings suggest that the pathology of growth faltering extends beyond the small intestine and may be more prominent in the large intestine.

## Results

### Cohort description and systemic markers of growth faltering and diarrhea

**Fig. 1:**
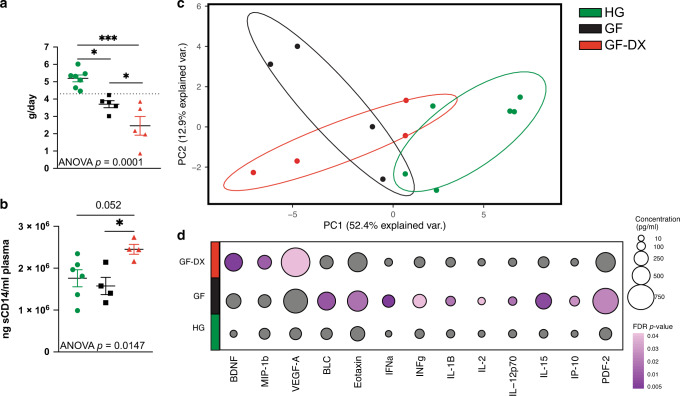
Growth faltering and growth faltering with active chronic diarrhea are associated with heightened but distinct systemic inflammation. **a** Growth rate (grams/day) was calculated for each infant in comparison with the colony average growth rates (calculated from 6510 rhesus macaques, split evenly between males and females) for the same period (dashed line). Animals that fell below the average growth rate were characterized as GF, animals below the average growth rate and with active diarrhea were classified as GF-DX, and animals above the average were considered HG. **b** Plasma levels of soluble CD14, a marker or systemic inflammation. **c** Principal component analysis of circulating immune mediators measured in plasma by multiplexed Elisa. **d** Bubble plots of circulating immune factor levels (picograms per milliliter of plasma). A total of 33 analytes were measured and only analytes significantly different by Kruskal Wallis non-parametric One-Way ANOVA (*p* < 0.05) were included in the plot. The size of each circle indicates the mean concentration of the indicated analyte and the color of the circle denotes the *p*-value of Dunn’s multiple comparisons test. Post-hoc comparisons were made between HG vs. GF and HG vs. GF-DX.

In this study, a total of 17 6–12-month old infant macaques were classified into three groups; Healthy growth (HG), Growth Faltering with acute distance or no history of diarrhea (GF), and Growth Faltering with active chronic diarrhea (GF-DX) based on growth rate and clinical status at the time of necropsy (Fig. 1a, Supp. Table 1). Briefly, HG (*n* = 7) grew at or above the average growth trajectory with only 1 of the 7 infants experiencing diarrhea ~3.3 months before necropsy (Supp. Table 1). In the GF (*n* = 5) group, 3 of 5 of the infants had experienced clinical diarrhea but had not had an episode of diarrhea for at least one 1-month before necropsy (Fig. 1a, Supp. Table 1). GF-DX (*n* = 5) all had a history of active chronic diarrhea and had diarrhea at the time of necropsy (Fig. 1a, Supp. Table 1). The clinical history of infants that experienced diarrhea was previously described^29^.

We next measured the levels of several systemic markers associated with barrier dysfunction and inflammation. We observed significantly higher levels of circulating soluble CD14 in GF-DX infants, with no differences between the GF and HG groups (Fig. 1b). No differences in circulating free or IgM bound LPS, or internal fatty acid-binding protein (iFABP) were observed between the three groups (Supp. Fig. 1a–c). Circulating levels of cytokines, chemokines, and growth factors were measured using multiplex ELISA (Luminex). A principal component analysis (PCA) revealed that the circulating immune mediator profile of both GF and GF-DX infants is distinct from that of the HG group (Fig. 1c). Levels of pro-inflammatory cytokines (Eotaxin, IFNα, IFNγ, IL-1β, IL-2, IL-15, and IL-12p70), as well as chemokines (BLC, IP-10, and PDF-2), were higher in GF compared to HG infants (Fig. 1d). On the other hand, levels of BDNF, MIP-1b, and VEGF-A were higher in GF-DX infants compared to HG infants (Fig. 1d). In addition, the levels of IL-8, IL-23, TNFα, VEGF-d were significantly different between the three groups by Kruskal Wallis non-parametric ANOVA, but not significantly elevated in a single group.

### Growth faltering is associated with altered immune cell frequencies

**Fig. 2:**
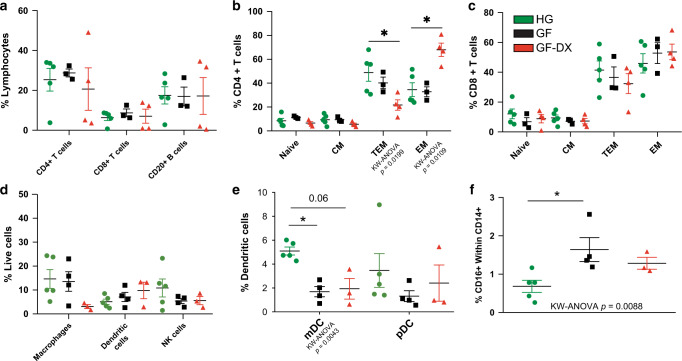
Growth faltering and growth faltering with active chronic diarrhea coincide with a unique immune cell population in the colon. **a** Scatter plots denoting percent abundance of lamina propria T and B cells in the colon. Naïve and memory T cell subsets of colonic lamina propria for (**b**) CD4+ and (**c**) CD8+ T cells. **d** Percent abundance of lamina propria dendritic cells (DC), macrophages, and natural killer (NK) cells in the colon along with (**e**) mDCs and pDC subsets, and (**f**) CD16+ Macrophages. Each point represents a study animal. Horizontal bars and whiskers indicate the mean +/− SEM. Significance was determined using Kruskal Wallis non-parametric ANOVA, with Dunn’s multiple comparisons *p < 0.05, **p < 0.01. Post-hoc comparisons were made between HG vs. GF and HG vs. GF-DX.

We next measured the frequency of several immune subsets in ileal and colonic LPLs using flow cytometry. The overall frequencies of T or B-lymphocytes within colonic LPLs did not differ significantly between the three groups (Fig. 2a). However, the frequency of colonic CD4+ effector memory (EM) T cells was significantly increased while that of CD4+ transitional effector memory (TEM) T cells was decreased in GF-DX animals (Fig. 2b) indicative of accelerated differentiation of colonic CD4 T cells in GF-DX infants (Fig. 2b). No differences were detected in the relative frequencies of naïve or memory colonic CD8+ T cell subsets (Fig. 2c). In the ileum, we observed a significant decrease in the abundance of total CD4+ T cells in GF-DX infants (Supp. Fig. 1d), but no differences were observed in ileal CD8+ T cells or B cells (Supp. Fig. 1d). No differences were observed in the frequency of ileal naïve and memory CD4 or CD8 subsets (Supp. Fig. 1e, f).

No differences were detected in the frequency of dendritic cells (DCs), macrophages, or NK cells at either gut site (Fig. 2d, Supp. Fig. 1g). The frequency of colonic myeloid dendritic cells (mDC) was decreased in GF animals and trended lower in GF-DX animals (Fig. 2e), while the frequency of activated colonic CD16+ macrophages increased in GF animals (Fig. 2f). No differences in the frequencies of these subsets were detected between the three groups in the ileum (Supp. Fig. 1h, i).

### Growth faltering with chronic active diarrhea results in dampened colonic LPL response to stimulation

**Fig. 3:**
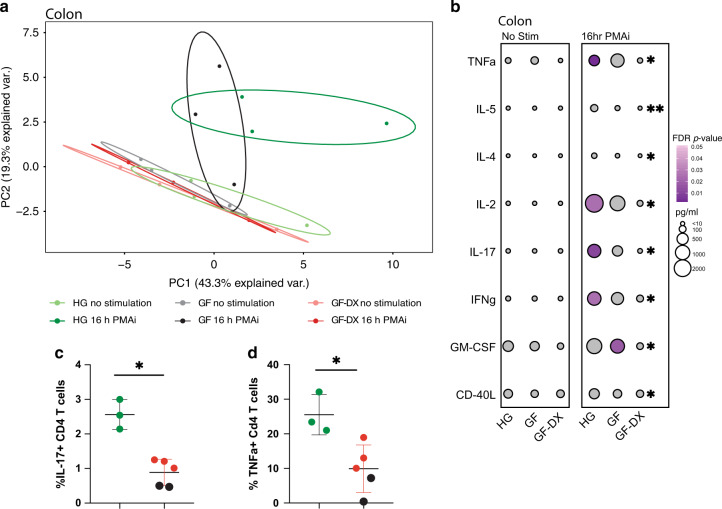
Growth faltering with active chronic diarrhea but not growth faltering alone disrupt immune mediator production by colonic LPLs. **a** The principal component analysis generated from the levels of 33 immune mediators released by colonic LPLs collected from HG, GF, and GF-DX infants in the absence and presence of PMA/ionomycin (PMAi) stimulation. **b** Bubble immune factor production (picograms per milliliter) in the presence or absence of PMAi stimulation by colonic LPLs. A total of 33 analytes were measured and only analytes significantly different by Kruskal Wallis non-parametric ANOVA (*p* < 0.05) were included in the plot. The size of each circle indicates the mean concentration of the indicated analyte and the color of the circle denotes the *p*-value of Dunn’s multiple comparisons test. Post-hoc comparisons were made between HG vs. GF and HG vs. GF-DX. **c**, **d** Percent of circulating CD4+ T cells producing (**c**) IL-17 and (**d**) TNFa after 6-h stimulation with PMAi. Horizontal bars and whiskers indicate the mean +/− SEM. Significance was determined using an unpaired *t*-test, *p > 0.05.

To explore the impact of growth faltering/diarrhea on the function of LPLs, cells were stimulated with PMA/ionomycin (PMAi) for 16 h and the production of 33 immune mediators was measured using Luminex. No significant differences in the production of immune mediators by ileal LPLs were detected between the three groups of infants with or without PMAi stimulation as illustrated by the overlap of samples in the PCA plot (Supp. Fig. 2a) and the absolute amounts measured in supernatants (Supp. Fig. 2b). However, PMAi stimulation resulted in the increased production of CD-40L and CCL5 in only the HG group (Supp. Fig. 2b). In contrast, immune mediator production by colonic LPLs in response to PMAi was quite distinct between the three groups (Fig. 3a). Specifically, while colonic LPLs from healthy and GF animals formed distinct clusters post-stimulation, those from GF-DX animals overlapped with the unstimulated samples (Fig. 3a). Indeed, the concentration of pro-inflammatory cytokines (e.g., TNFα, IL-17, IFNγ), T cell-specific factors (e.g., IL-2, IL-4, IL5, CD-40L), and the growth factor GM-CSF were significantly reduced compared to those observed from the HG (Fig. 3b). To determine if this defect in immune activation was limited to the gut, PBMC were stimulated with PMAi for 6 h, and cytokine production measured by intracellular cytokine staining. Due to the paucity of available samples, the GF and GF-DX groups were combined for this experiment. As described for LPLs, the frequency of CD4 T cells secreting IL-17 and TNFα was significantly reduced in GF/GF-DX animals (Fig. 3c, d).

**Fig. 4:**
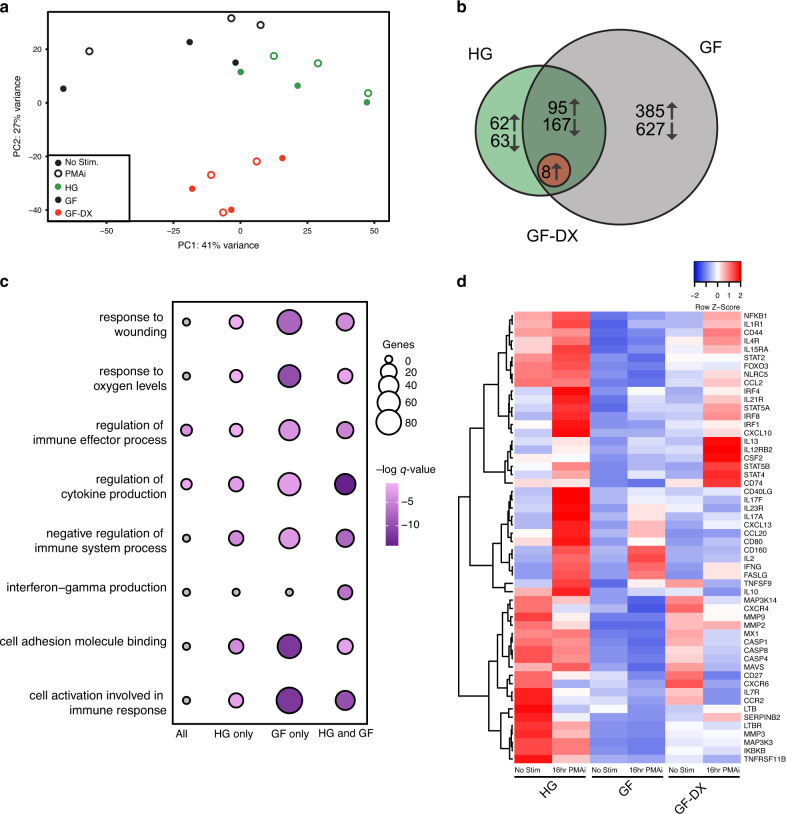
Transcriptional changes of colonic Lamina propria leukocytes (LPLs) in response to PMAi stimulation. **a** Principal component analysis of transcriptomic data from colonic LPLs in the absence and after PMA/ionomycin stimulation for 16 h from HG, GF, and GF-DX infants. **b** Venn diagrams of DEGs detected in colonic LPLs obtained from HG, GF, and GF-DX infants using in response to stimulation using pairwise EdgeR analysis. **c** Functional enrichment of DEG detected in the colon and ileum of infants determined using Metascape. Each circle denotes a gene ontology (GO) term. The size of the circle reflects the number of DEGs that enriched to that GO term. **d** Heatmap of select DEGs from the “cell activation involved in immune response” and “interferon-gamma production” GO-terms normalized by row.

We further explored the impact of growth faltering/diarrhea on colonic LPLs response to PMAi using RNA-seq. PCA shows that the transcriptional profile of the 3-groups was distinct regardless of stimulation and that LPLs from GF-DX animals are distinct from those from HG and GF animals (Fig. 4a). Pairwise differential gene expression (DEG) showed a robust transcriptional response to PMAi stimulation with 387 and 1274 DEGs in HG and GF infants, respectively (Fig. 4b). In contrast, only 8 DEGs were detected in colonic LPLs from GF-DX animals in response to stimulation (Fig. 4b). Functional enrichment using Metascape revealed that PMAi-induced DEGs in HG and GF LPLs enriched to gene ontology (GO) terms associated with a response to immune stimulation such as “cell activation involved in an immune response”, “regulation of cytokine production” and “negative regulation of immune system processes” (Fig. 4c). In agreement with our cytokine measurements, *IL-17A*, *CD40LG*, *IL2*, and *IFNg* were highly expressed in colonic LPLs from HG animals and to a lesser extent from GF animals after stimulation (Fig. 4d). Moreover, *IL10* and *CD74* were upregulated in response to stimulation in HG and GF infants but downregulated in infants with acute diarrhea (Fig. 4d).

### Gut transcriptional response to GF and GF-DX

**Fig. 5:**
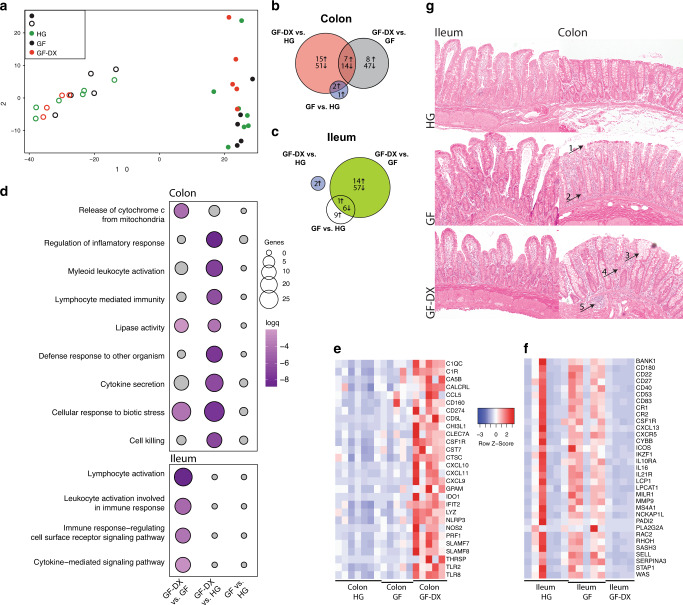
Transcriptional and histological changes in ileal and colonic gut biopsies from animals experiencing growth faltering and/or active chronic diarrhea. **a** Principal component analysis of transcriptomic data generated from ileal and colonic biopsies of healthy, faltering and faltering with diarrhea infants. **b** Venn diagrams of DEGs detected in colonic tissue biopsies obtained from HG, GF, and GF-DX infants. **c** Venn diagrams of DEGs detected in ileal tissue biopsies obtained from HG, GF, and GF-DX infants. **d** Functional enrichment of DEG detected in the colon and ileum of infants determined using Metascape. Each circle denotes a gene ontology (GO) term. The size of the circle reflects the number of DEGs that enriched to that GO term. **e**, **f** Heatmaps of select DEGs upregulated in the colon of GF-DX animals (**e**) and the ileum of GF animals (**f**) normalized by row. **g** Representative hematoxylin and eosin-stained images of the ileum and descending colon captured at ×10 magnification by a blinded pathologist. Arrows indicate hallmarks of intestinal inflammation: (1) epithelial disruptions, (2) increased lamina propria immune cells and elongated crypts, (3) crypt distortion, (4) crypt abscess, and (5) increased immune infiltration in the sub-mucosa.

To elucidate the effects of growth faltering/diarrhea on gut tissue homeostasis, we compared the transcriptomes of ileal and colonic tissue biopsies obtained from the three groups. PCA showed that the gut site exerted the most significant impact on the overall transcriptional profiles (Fig. 5a). DEG analysis between groups within colonic biopsies resulted in the identification of 87 DEGs between GF-DX and HG infants, 76 DEGs between GF-DX and GF infants, and only 3 DEGs between GF and HG infants (Fig. 5b, Supp. Table 2).

Functional enrichment using MetaScape showed that colonic DEGs unique to GF-DX vs. HG comparison enriched to GO terms associated with inflammation such as “regulation of inflammatory response”, “cytokine secretion”, and “lymphocyte-mediated immunity” (Fig. 5d). DEGs that enriched these GO terms were highly expressed in the colon of GF-DX infants (Fig. 5e) including *TLR2*, *TLR8*, *LYZ*, *IDO1*, *CXCL10*, and *CCL5* (Fig. 5e). DEGs unique to the GF-DX vs. GF comparison enriched to the GO-term “release of Cytochrome C from the mitochondria” a key step in apoptotic cell death (Fig. 5d). Finally, we used IMMQUANT to infer cell populations based on transcriptional profiles. In agreement with our Flow cytometry experiment, this analysis predicted an increase in macrophages in GF infants, as well as a decrease in the frequency of early B cells and pro-B cells within the colons of GF and GF-DX infants, respectively relative to HG infants (Supp. Fig. 2c).

In the ileum, only 2 DEGs were identified between GF-DX and HG, 78 DEGs between GF-DX and GF infants, and 16 DEGs between GF and HG infants with 7 common DEGs (Fig. 5c). DEGs unique to GF-DX vs. GF infants enriched to immunological GO terms including “lymphocyte activation” and “cytokine-mediated signaling pathway” (Fig. 5d). DEGs enriching to these GO terms were highly expressed in the ileal biopsies of GF infants and included; *IL10RA*, *ICOS*, *CD27*, *CXCR5*, and *CXCL13* (Fig. 5f). IMMQUANT analysis of ileal expression profiles predicts a decrease in effector memory CD8+ T cells in GF infants along with a decrease in granulocytes and an increase in plasmacytoid dendritic cells in GF-DX infants (Supp. Fig. 2d).

Since our RNA seq data suggested a significant increase in tissue inflammation in the colon with minimal changes in the ileum, we next evaluated inflammatory changes through histopathology. Examination of H&E stained slides from the mi-ileum revealed no gross differences between all three groups (Fig. 5g). Limited villous blunting and uniform intraepithelial lymphocyte infiltration was noted in all three groups (Fig. 5g). In contrast, analysis of the descending colon revealed significant damage in both the GF and GF-DX animal including crypt abscessations, inflammation in the submucosa, crypt hyperplasia, and neutrophil infiltration, all of which were most pronounced in the GF-DX animal (Fig. 5g).

### Growth faltering and diarrhea are associated with dysbiosis and loss of compartmentalization of the intestinal microbiome

**Fig. 6:**
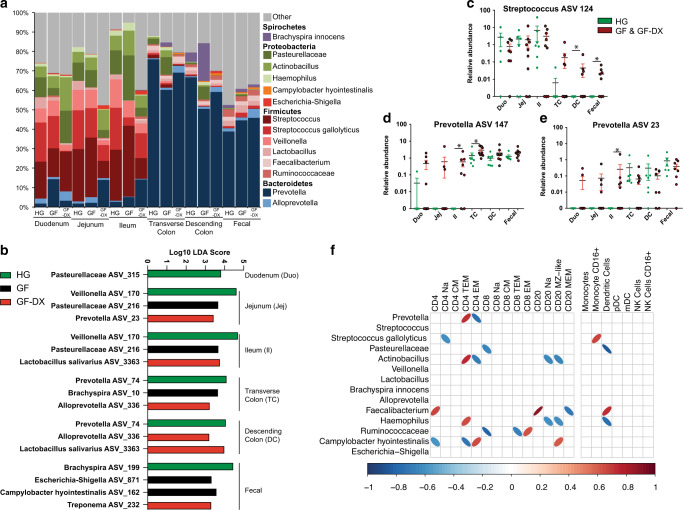
Luminal microbial communities of the small and large intestine are distinct and shift with growth faltering and/or active chronic diarrhea. **a** Stacked bar plot organized by sampling site and host status. All taxa below 1% average abundance grouped into the “Other” category. Bars represent the average for the indicated sample site/status. **b** Select differentially abundant ASVs between HG, GF, and GF-DX infants at each gut site. Differential abundance was determined using LEFsE (Log_10_ LDA score >2). **c**–**e** Scatter plots of ASVs that displayed differential abundance patterns across gut sites between HG and combined GF and GF-DX infants. The unpaired *t*-test between HG and combined GF and GF-DX infants at each site, *p < 0.05, **p < 0.01, ***p < 0.001. **f** Correlation analysis between colonic lamina propria immune cell populations and microbial taxa with >1% average abundance. Only correlations with uncorrected *p* < 0.05 are shown as ellipses, with blue indicating a negative correlation and red a positive. The width of each ellipse is proportional to the strength of the correlation with a narrower ellipse indicating a lower *p-*value.

We next interrogated the association of growth faltering/diarrhea with the taxonomic landscape of the luminal microbiome across multiple gut sites (duodenum, jejunum, ileum, traverse colon, descending colon) and fecal samples obtained from the same HG, GF, and GF-DX infants using 16S rRNA gene amplicon sequencing. Gut site, rather than host status or individual, was the major determinant of community composition for weighted UniFrac dissimilarity (54% of total variation) with microbial communities of the small intestine (duodenum, jejunum, and ileum) clustering separately from those of the colon and fecal materials (Supp. Fig. 3a, b). Nevertheless, we observed an increase in phylogenetic diversity in jejunal and ileal communities from GF-DX infants compared to those from HG infants (Supp. Fig. 3c). Regardless of host status, the luminal microbiome of the small intestine was dominated by *Streptococcus*, Pasteurellaceae, *Actinobacillus*, and *Veillonella*; while *Prevotella* was the most abundant taxa in the large intestine and fecal samples (Fig. 6a).

Next, we determined which bacterial taxa were differentially abundant between healthy and faltering infants at each gut site. Only amplicon sequence variants (ASVs) detected in at least two samples at greater than 0.01% relative abundance were included in this analysis (Fig. 6b, Supp. Table 3). A total of 75 ASVs were differentially abundant across all gut sites (1 Duodenum, 8 Jejunum, 17 Ileum, 23 Transverse Colon, 13 Descending Colon, 13 Fecal) (Supp. Table 3). Notable differences include a higher abundance of multiple Pasteurellaceae, *Lactobacillus*, and *Veillonella* ASVs in the small intestine luminal contents from HG infants (Fig. 6b, Supp. Table 3). Similarly, small intestine luminal contents of GF infants were enriched in multiple Pasteurellaceae ASVs, while those from GF-DX infants had a higher abundance of ASVs assigned to *Prevotella*, *Catenibacterium*, and *Lactobacillus salivarius* (Fig. 6b, Supp. Table 3). The large intestine and fecal microbiome of HG infants were enriched in multiple *Prevotella* and Ruminococcaceae ASVs, as well as *Faecalibacterium* and *Bracyspira* (Fig. 6b, Supp. Table 3). On the other hand, the colonic microbiome of GF-DX infants was enriched in *Lactobacillus salivarius* along with multiple different *Prevotella* and Alloprevotella ASVs (Fig. 6b, Supp. Table 3). Finally, the fecal microbiome of GF infants was enriched in ASVs assigned to *Campylobacter hyointestinalis*, and *Escherichia-Shigella* (Fig. 6b, Supp. Table 3).

Growth faltering and diarrheal diseases are hypothesized to lead to a decompartmentalization of the gut microbiome; however, this pattern has only been inferred and has not been observed directly using paired samples. Fecal samples collected from GF/GF-DX animals had significantly lower observed ASVs and more intra-group community variability at all sights except the duodenum (Supp. Fig. 3d,e). We next plotted the abundance of taxa enriched in the luminal microbiomes of GF/GF-DX compared to HG infants along the GI tract (Fig. 6c–e, Supp. Fig. 3f–h, Supp. Table 4). This analysis showed that *Streptococcus* ASV 124, which is normally relegated to the small intestine luminal microbiome in HG animals, was abundant in the large intestine and fecal microbiome of GF/GF-DX (Fig. 6c). Similarly, the relative abundance of several ASVs normally relegated to the large intestine in HG animals (*Prevotella* ASV 147 and 23, *Cantenibacterium* ASV 156, and Lachnospiraceae ASV 24) was higher in the small intestine of GF/GF-DX infants (Fig. 6d,e, Supp. Fig. 3g,h). Finally, *Alloprevotella* ASV 336 was found almost exclusively in the lumen of GF/GF-DX infants and was significantly more abundant in both colon sections (Supp. Fig. 3f).

We next explored associations between immune dysregulation and microbial dysbiosis in GF and GF-DX animals. In the traverse colon, we observed 25 significant (*p* < 0.05) correlations between immune cell populations and the luminal microbiome (Fig. 6f). These correlations included positive correlations between *Prevotella* and CD4+ TEM T cells, *Faecalibacterium,* and total CD20+ B cells, along with *Campylobacter hyointestinalis* and CD4+ EM T cells (Fig. 6f). On the other hand, Pasteurellaceae and Ruminococcaceae were negatively correlated with total DC and CD8+ T cells, respectively (Fig. 6f). In the Ileum, we observed 8 significant correlations between local immune cell populations and the microbiome (Supp. Fig. 3i). These included positive correlations between total Macrophages and *Lactobacillus*, and a negative correlation between *Alloprevotella* and total CD8+ T cells (Supp. Fig. 3i).

### Growth faltering and diarrhea are associated with functional shifts in the luminal microbiome

To enhance the resolution at which microbial communities are defined, we utilized shotgun metagenomics. More reads mapped to the host genome in the ileal luminal contents compared to colonic luminal contents due to the lower microbial biomass found in the small intestine (Supp. Fig. 4a, Supp Table 5). At the Phyla level, colonic luminal contents had significantly more Bacteroidetes, Spirochetes, Actinobacteria, and Euryarchaeota than ileal luminal contents (Supp. Fig. 4b, Supp Table 6). Reads that mapped to microbial taxa or functional genes in the ileum were distinct from those obtained from the colon regardless of host health status (Supp. Fig. 4c, d). A total of 46 species were differentially abundant between the ileum and the colon (Supp Table 6). Notably, *Lactobacillus amaylovorus* and *Lactobacillus mucosae* were enriched in the ileum while *Prevotella copri*, *Helicobacter macacae*, and *Treponema succinifaciens* were enriched in the colon (Supp Table 6).

Next, we compared the abundance of taxa between groups. The ileal luminal contents from HG infants were enriched in *Lactobacillus reuteri* while those of GF-DX infants had a higher abundance of *Lactobacillus salivarius* (Supp. Fig. 4e). Larger differences between the three groups were observed in the colonic luminal contents. Microbial communities from HG infants had a higher abundance of the commensals *Helicobacter macacae* and *Ruminococcus obeum* (Supp. Fig. 4g). In contrast, microbial communities of GF and GF-DX are enriched in potential pathobionts notably *Fusobacterium nucleatum* and *Helicobacter cinaedi* in GF infants (Supp. Fig. 4g) and *Lactobacillus salivarius, Escherichia coli*, and *Enterococcus faecalis* in GF-DX infants (Supp. Fig. 4g).

Although the overall functional potential of the ileal contents did not significantly differ between the three groups as measured by Bray Curtis dissimilarity (permanova *p* > 0.05) (Supp. Fig. 4d), the ileal microbiome of HG infants was enriched in the pathway for stachyose degradation, folate transformation and galactose degradation (Supp. Fig. 4f). The ileal microbiome of GF infants was enriched in pathways for L-histidine degradation III and D-beta-fucofuranose biosynthesis, while that of GF-DX infants were enriched for L-methionine and L-ornithine biosynthesis (Supp. Fig. 4f). Similarly, the overall functional potential of colonic luminal contents did not differ significantly between HG and GF infants as measured by Bray Curtis dissimilarity (permanova *p* > 0.05) (Supp. Fig. 4d). Nevertheless, the colonic microbiome of HG infants was enriched for pyruvate fermentation to butanoate, pyruvate fermentation to hexanol, and glutaryl-CoA degradation pathways (Supp. Fig. 4h), while those of GF infants were enriched in the citrulline biosynthesis pathway, and those of infants with GF-DX in pathways for thiazole biosynthesis, fatty acid degradation, and ornithine degradation (Supp. Fig. 4h).

**Fig. 7:**
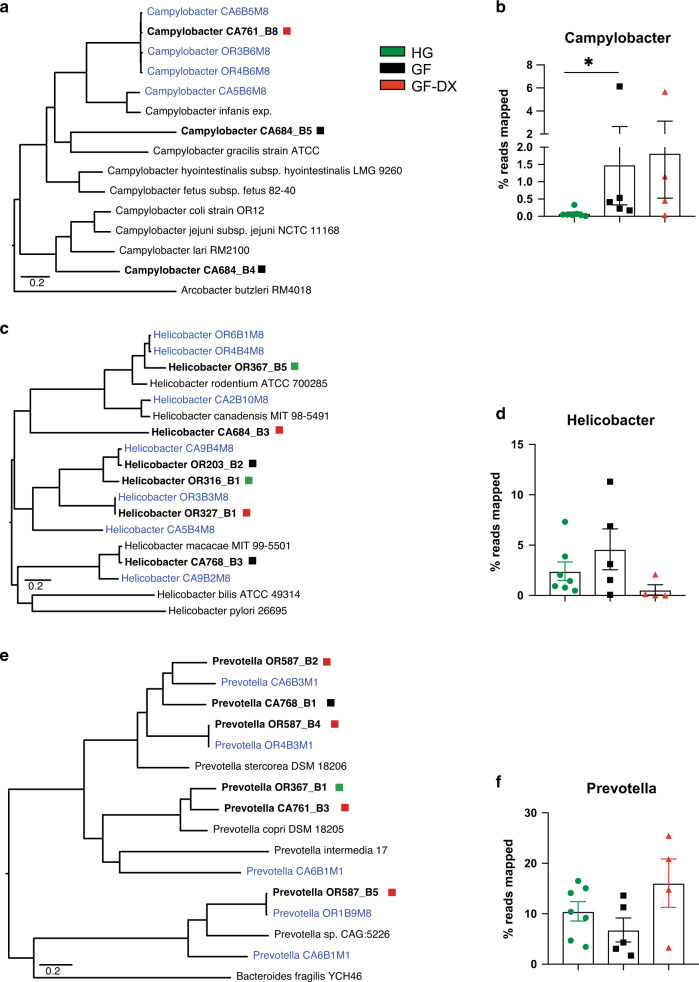
Genome assembly reveals a higher abundance of Campylobacter in growth faltering infants. **a***Campylobacter* core genome phylogram built on the alignment of all protein-coding genes common to all members of the tree (4 assembled genomes from colonic luminal contents [Bold], 4 genomes previously assembled from infant Rhesus macaque feces [Blue], 7 Published *Campylobacter* genomes [Black]) with exception of the outgroup *A. butzerli*. **b** Percentage of metagenomic reads that align to the 3 assembled Campylobacter genomes cumulatively. (Mann-Whitney test; **P < 0.01). **c** *Helicobacter* core genome phylogram built on the alignment of all protein-coding genes common to all members of the tree (6 assembled genomes from colonic luminal contents [Bold], 7 genomes previously assembled from infant Rhesus macaque feces [Blue], and 4 Published Helicobacter genomes) with exception of the outgroup *H. pylori*. **d** Percentage of metagenomic reads that align to the 6 assembled Helicobacter genomes cumulatively. (Mann-Whitney test; **P < 0.01). **e** *Prevotella* core genome phylogram built on the alignment of all protein-coding genes common to all members of the tree (6 assembled genomes from colonic luminal contents [Bold], 5 genomes previously assembled from infant Rhesus macaque feces [Blue], 4 Published *Prevotella* genomes [Black]) with exception of the outgroup *B. fragilis*. **f** Percentage of metagenomic reads that align to the 9 assembled *Prevotella* genomes cumulatively. (Mann-Whitney test; **P < 0.01).

We were able to assemble 23 putative microbial genomes from colonic luminal contents with >80% completeness and <2% contamination as measured by checkM^39^. including 9 *Prevotella*, 6 *Helicobacter*, and 3 *Campylobacter* genomes (Supp. Table 7). The three assembled *Campylobacter* genomes were distinct from each other and most closely related to *Campylobacter garcilis, Campylobacter coli/jejuni*, and the recently proposed species, *Campylobacter infanis*^25^. (Fig. 7a). Importantly, all *Campylobacter* genomes were only assembled from GF and GF-DX infants, and significantly more reads mapped to these assembled genomes in samples from GF and GF-DX infants compared to HG infants (Fig. 7b). Helicobacter genomes were assembled from all three groups (Fig. 7c) and no differences were noted in the percentage of reads mapping to these genomes between the three groups (Fig. 7d). In addition, we assembled a diverse set of *Prevotella* genomes primarily from GF infants (Fig. 7e); however, no differences in the abundance of reads that mapped to these genomes were observed between the groups (Fig. 7f).

## Discussion

In addition to high mortality, diarrheal diseases are a major driver of malnutrition, as well as growth and cognitive stunting^4,6,13,40,41^. One of the major knowledge gaps in the field is the lack of studies pertaining to host-microbial interactions within the gut. These studies are difficult to conduct in humans due to challenges associated with obtaining samples along the entire GI tract. Rhesus macaques are naturally susceptible to human enteric pathogens that result in diarrhea and growth faltering^36,37^. In this study, we leveraged this translational model to elucidate the immunological and microbial changes associated with growth faltering and a history of diarrhea throughout the GI tract in addition to fecal samples.

In contrast to what has been reported in humans^42,43^ we did not observe changes in systemic levels of molecules associated with barrier dysfunction (iFABP) or bacterial translocation (LAL, IgM bound endotoxin) in GF and GF/DX infants. However, our analysis revealed higher levels of systemic inflammatory markers in both groups of growth faltering infants. In addition, we found a stark increase in the levels of BDNF which was detectable in 2/6 HG infants, 3/4 GF infants, and 4/4 GF-DX infants. BDNF can be produced by multiple cell types in the gut^44,45^ and is important for the regulation of tight junctions^46^. Moreover, systemic levels of BDNF have been positively correlated with visceral hypersensitivity (pain) in irritable bowel syndrome (IBS)^45,47^. Our data suggest that plasma levels of BDNF could be used as an alternative marker of intestinal injury. Interestingly, plasma levels of IL-1β, IFNα, IFNγ, and IL-15 were higher in GF infants, indicating that distant history of diarrhea can have long-lasting ramifications on the host immune status. High levels of systemic IL-1β have been linked to poor neurodevelopment in Bangladeshi children^48^, while IFNγ has been associated with increased microbial translocation in growth faltering infants^49^. This enhanced systemic inflammation was accompanied by a dampened IL-17 and TNFα response of peripheral CD4 T cells to polyclonal stimulation, potentially an indication of an immunoregulatory phenotype.

Significant differences in immune cell frequencies and response to polyclonal stimulation between HG and GF-DX animals were observed in colonic, but not ileal, LPLs. Specifically, we observed a reduction in the relative frequency of colonic mDCs, a major antigen-presenting cell subset^50^, in both faltering groups. This loss could be one of the factors contributing to the reduced immune response to oral Rotavirus and Polio vaccines observed in children with growth faltering and diarrheal diseases^51,52^. In addition, we detected a higher relative frequency of the non-classical colonic CD16+ macrophages in GF infants, indicative of higher inflammation even in the absence of active diarrhea. Indeed, CD16+ macrophages in the gut have been shown to mediate fibrosis in inflammatory bowel disease^53^. We also observed an increase in the percentage of terminally differentiated colonic memory CD4+ T, suggesting an accelerated differentiation. CD4+ T cells are the most abundant LPL population and play important roles in tissue maintenance and anti-microbial responses^54–56^. In healthy infants, tissue-resident T cells are typically naive, and regulatory and resident effector memory cells do not develop until adulthood^57,58^. High pathogen load or repeated exposure to antigen could drive the observed shifts in memory CD4 T cell populations. Terminally differentiated memory T cells are usually associated with increased inflammatory responses. However, colonic LPLs from GF-DX animals generated a dampened response to polyclonal stimulation relative to colonic LPLs from HG infants failing to produce canonical cytokines IL-2, IL-17, and IFNγ. This dampened response was also evident at the transcriptional level. Interestingly colonic LPLs from GF infants generated a larger transcriptional response to polyclonal stimulation in line with increased plasma levels of inflammatory cytokines. Together these observations suggest that GF with a limited diarrhea history is associated with exacerbated inflammation while GF with chronic diarrhea is associated with immune exhaustion. However, further measurement of exhaustion markers on LPL such as PD-1 and CTLA-4 in the colon is needed to confirm this.

Our transcriptomic analysis of ileal and colonic biopsies revealed the downregulation of genes important for immune activation and highly expressed by gut-associated B cells^59^ in GF-DX compared to GF infants. This suggests that GF in the absence of active diarrhea is associated with increased B cell activity in the ileum. B cells have been shown to increase in abundance and aggregate in the small intestine of growth faltering infants in the Gambia^49^. This chronic immune activation may contribute to systemic inflammation and inhibit the host from adequately responding to mucosal vaccines/infection. In contrast, the transcriptional landscape in the colon was indicative of enhanced immune activation in GF-DX infants compared to GF infants. Many of the genes upregulated in the colon of GF-DX animals were associated with T cell and macrophage activation in agreement with our flow cytometry data, as well as granulocytes (not included in our immunophenotyping). Finally, histological examination of tissue biopsies from the mid-ileum and traverse colon confirmed that GF is associated with a heightened inflammatory landscape in the colon. Taken together, our phenotypic, functional, transcriptional, and histological data strongly suggest that GF, especially when coupled with chronic active diarrhea, primarily impacts the colon rather than the small intestine. While more recent human studies have focused on the small intestine, earlier studies identified mucosal abnormalities in rectal biopsies of volunteers in southern India which they termed tropical colonopathy^60^.

This study provided the first insight into the microbial communities within luminal contents along the intestinal tract of infant macaques. As previously reported for adult macaques^61^, the luminal microbiome of the small and large intestines are distinct. Phylogenetic diversity in the small intestinal was higher in GF-DX infants. On the other hand, and as described in human infants with, malnutrition^32^ and growth faltering^31^, the alpha diversity in the fecal samples of GF/GF-DX macaques was reduced compared to HG macaques. Furthermore, inter-sample variability was higher in microbial communities from GF/GF-DX animals, indicative of microbiome instability or a more immature state as has been shown in the fecal microbiome of malnourished children^32^.

In addition, multiple bacterial taxa normally sequestered to either the small or large intestine in HG infants were detected in several gut sites in GF/GF-DX infants, suggesting the loss of strict compartmentalization between gut-sites with growth faltering. Specifically, the relative abundance of *Lactobacillus salivarius*, a core member of the oral microbiome in humans^62^, were increased in the lumen of both the small and large intestine of GF-DX infants. *L. salivarius* has been reported to be enriched in the fecal samples of growth-stunted children^33^. Moreover, *Streptococcus*, also part of the core oral microbiome^62^ and normally relegated to the small intestine in healthy animals, was detected in colonic luminal contents. This observation is similar to recent reports describing the detection of nasopharyngeal taxa to the gut in children experiencing growth stunting^33^. This loss of strict compartmentalization could be due to a reduced ability of the host immune system to tightly control microbial growth.

We also observed differences in the functional potential in both the ileal and colonic luminal microbiome. Specifically, we observed a reduced capacity of short-chain fatty acid (SCFA) production in GF and GF-DX infants in both ileal and colonic microbial communities. The microbial production of SCFA has been closely linked to the activation of mucosal Th1, Th17, and Tregs^63^. However, SCFA has also been reported to skew T cells towards anti-inflammatory IL-10 production and play a role in preventing colitis and other inflammatory conditions^64^. In addition, the SCFA butyrate can modulate the function of innate immune cells by reducing the expression and nuclear translocation of NF-κB in intestinal macrophages^65,66^. Therefore, the loss of SCFA production potential in GF and GF-DX animals may contribute to the heightened inflammatory state we observed.

We also observed correlations between intestinal immune cell frequencies and the relative abundance of microbes. In the Ileum this included a positive correlation between *Lactobacillus* abundance and Ileal Macrophages. *Lactobacillus* strains have also been shown to activate macrophages in the small intestine^67,68^. The increased abundance of *Lactobacillus salivarius* in the small and large intestine of GF-DX infants coupled with increased frequency of activated macrophages suggest an infiltration of oral microbes into the small intestine resulting in activation of the innate immune system. In the colon, we found an inverse relationship between *Prevotella* and *Campylobacter hyointestinalis* with colonic CD4+ T cell subsets. While gut *Prevotella* has been linked to systemic inflammation and reduced systemic CD4+ T cells in the western world, particularly in HIV infection^69–71^. It is important to note that *Prevotella* is the most abundant microbe in the gut of humans with a non-westernized lifestyle and is largely believed to be beneficial in this context^72,73^. However, the positive correlation of CD4+ EM T cells with *Campylobacter hyointestinalis* may indicate recurrent infection and the expansion of a pathogen-specific T cell subset.

We detected an increased abundance of *Campylobacter hyointestinalis* and *Escherichia-Shigella* in fecal samples collected from GF infants, indicative of an increased pathogen burden, as reported in multiple human studies^19,74,75^. Diversity within the *Campylobacter* genus is often missed in 16S amplicon surveys due to their highly conserved 16S rRNA gene^76^; therefore, we resorted to metagenomic genome assembly. We constructed 3 *Campylobacter* genomes only from the colonic luminal contents of faltering infants. Moreover, a higher number of short reads mapped to these three genomes in colonic luminal contents of faltering infants. One of our assembled *Campylobacter* genomes, as well as some of our previously described^29^. *Campylobacter* assemblies from infant rhesus macaque feces were most closely related to the newly described “*Candidatus Campylobacter infanis*”^25^. Non-coli/jejuni *Campylobacter* has recently been implicated in diarrheal diseases and childhood mortality in the developing world^25,26,77^.

In contrast, we did not observe differences in the abundance of our assembled *Helicobacter* genomes. These data differ from our recent report of a decreased abundance of *Helicobacter* in the fecal microbiome of infants that experienced diarrhea and received antibiotics. This difference could be potentially be explained by the fact that *Helicobacter* is tightly associated with gut mucosa and greatly reduced in the lumen^61^. We also assembled several new *Prevotella* genomes mostly from GF animals. These new genomes differed from the cluster of *Prevotella* genomes we recently identified from the fecal microbiome of 1-month old infants that would later experience diarrhea^29^. Interestingly, the frequency of reads mapping to these genomes did not differ between the three groups even though 90% of the genomes were assembled from GF/GF-DX animals.

In summary, this study provides the first comprehensive look at the association of growth faltering and diarrheal disease with luminal microbiomes and local immune dysfunction that would not be possible in humans. Together, our data suggest that the colon is the major site of immune dysfunction during active diarrhea. We also found that immune dysfunction in GF and GF-DX is associated with a decompartmentalization of the luminal microbiome. Data presented in this manuscript highlight the need for novel multifaceted approaches that can address the complex etiology of GF and diarrheal disease with the goal of reducing pathogen burden, restoring gut integrity, and rejuvenating the intestinal immune system. Previous interventions aimed at reducing childhood mortality due to growth faltering and enteric disease^78^ including the prophylactic administration of azithromycin^77,79^, micronutrients^80^, probiotics (*Lactobacillus* species)^81^. However, many of these interventions have resulted in little to no improvement in growth or other primary endpoints^78^. Potential new strategies could include broad-acting phage therapy against *Campylobacter*, probiotic treatment with commensals that are relevant to children who live in the developing world, SCFA supplementation to reduce inflammation and improve gut immune fitness, and immune-modulatory drugs that can improve gut integrity. Infant macaques provide a highly relevant translational animal model to test these novel interventions and evaluate their impact on tissue inflammation, immune function, and microbial communities across all sections of the gut.

## Methods

### Cohort description

Samples were obtained from a total of 17 6–12-month old infant macaques (Supp. Table 1). All rhesus macaque studies were overseen and approved by the OHSU/ONPRC and University of California-Davis/CNPRC Institutional Animal Care and Use Committees’ (IACUC) per the National Institutes of Health guide for the care and use of laboratory animals. Animals were housed per the standards established by the US Federal Animal Welfare Act and *The Guide for the Care and Use of Laboratory Animals*. All animals were tested for simian viruses (Simian Immunodeficiency Virus, Simian Retrovirus 2, Macacine herpesvirus 1, and Simian T lymphotropic virus) and received a tuberculin test semi-annually.

### Measurement of systemic markers

Levels of Endotoxin-core antibodies IgM (Hycult Biotech, Uden, Netherlands), soluble CD14 (sCD14) (R&D Systems, Minneapolis, MN, United States), and internal fatty acid-binding protein (iFABP) (MyBioSource, San Diego, CA, United States) in plasma samples were measured using an enzyme-immunoassay technique (ELISA) following the manufacturer’s protocol. Plasma levels of free bacterial endotoxin were measured using a chromogenic Limulus amebocyte lysate assay (Hycult Biotech, Uden, Netherlands) following the manufacturer’s protocol. Plasma levels of circulating immune factors were measured using a Magnetic 33-plex assay (R&D Systems, Minneapolis, MN, United States) and a MAGPIX instrument (Luminex, Austin, TX, United States). Bubble plots were generated using ggplot2 R package with concentration (pg/ml).

### Isolation of lamina propria leukocytes (LPL)

Tissue biopsies were collected from the ileum and descending colon into Roswell Park Memorial Institute medium supplemented with 10% fetal bovine serum, streptomycin/penicillin, and L-glutamine (RP10). Lamina propria leukocytes (LPLs) were isolated using collagenase IX/DNAse digestion followed by centrifugation over a Percoll gradient (GE Healthcare, Waukesha, WI, USA) as previously described^82^. Cells were cryopreserved in FetalPlex Animal Serum Complex (Gemini Bio-Products, West Sacramento, CA, USA) and DMSO.

### Flow cytometry

1 × 10^6^ LPLs were stained using antibodies against CD3, CD4, CD8b, CD95, CD28, CCR7, and CD20 to delineate naïve and memory CD4 and CD8 T cells, as well as CD20 B cells. The cells were subsequently fixed and permeabilized before the addition of anti-Ki-67 to measure proliferation. T cells were then divided into Naive (CD28+, CD95−; Naive), central memory (CCR7+ CD28+ CD95+; CM), transitional effector memory (CCR7− CD28+ CD95+; TEM), and Effector memory (CCR7− CD28− CD95+; EM), and. The second tube of 1 × 10^6^ LPLs was stained using antibodies against: CD3, CD20, HLA-DR, CD14, CD11c, CD123, CD16, and CD8a to delineate macrophages (CD3− CD20− CD14 + HLA-DR+), dendritic cells (DC, CD3− CD20-CD14− HLA-DR+), and natural killer (NK, CD3-CD20-CD14− HLA-DR− CD8a+) cell subsets. Macrophages were further subdivided into classical (CD16−) and non-classical macrophages (CD16+). DCs were further subdivided into myeloid DC (mDC, CD123− CD11c+) and plasmacytoid DC (pDC, CD123+CD11c−;). All flow cytometry samples were acquired using Attune NxT (Life Technologies, Carlsbad, CA, United States) and analyzed using FlowJo (TreeStar, Ashland, OR, United States).

### LPL stimulation

LPLs (5 × 10^5^/well) were thawed and incubated for 16 h in the absence (unstimulated) or presence (stimulated) of 5 ng/ml PMA and 1 μg/ml Ionomycin (Sigma-Aldrich, St. Louis, MO, United States) at 37 °C in a humidified incubator (5% CO2). At the end of the incubation, cells were centrifuged for 5 min at 2000 rpm. Supernatants were collected to measure the production of immune mediators using a Magnetic 33-plex assay (R&D Systems, Minneapolis, MN, United States) and a MAGPIX instrument (Luminex, Austin, TX, United States). Absolute change of immune mediators was calculated by subtracting the basal level of production from the level post-stimulation in a pairwise fashion. Bubble plots were generated using ggplot2 R package with concentration (pg/ml).

### RNA-sequencing and data analysis

Total RNA was isolated from ileum and colon biopsies, as well as ileal and colonic LPLs following overnight culture in the presence/absence of PMA/Ionomycin using Qiagen miRNeasy Kit. RNA concentration and quality were verified using Agilent 2100 Bioanalyzer. RNA-seq libraries were constructed using the TruSeq Stranded Total RNA Kit (Illumina, San Diego, CA, United States). rRNA-depleted RNA was fragmented and converted to double-stranded cDNA. Adapters were ligated, and ~300 base pair fragments were amplified by PCR and selected by size exclusion using Ampure beads XP (Beckman Coulter, Brea, CA). Each library was labeled with a unique barcode for multiplexing. To ensure proper sizing, quantitation, and quality before sequencing, libraries were analyzed on the Agilent 2100 Bioanalyzer. Multiplexed libraries were sequenced (100 base pair single-end) using the HiSeq 4000 platform.

Data analysis was performed with the RNA sequencing workflow module of the systemPiperR package available on Bioconductor^83^. Quality filtration and trimming were performed using Trimmomatic with an average phred score cutoff of 30 and a minimum length of 75 bp. Reads were mapped with the splice-aware aligner suite Bowtie2/Tophat2 against the *Macaca mulatta* genome (Macaca_mulatta.MMUL_1.78). Raw count values were generated with summarizeOverlaps function. Only reads overlapping exonic regions of genes were counted, discarding reads mapping to ambiguous regions of exons from overlapping genes. Analysis of differentially expressed genes (DEGs) was performed using edgeR package^83^. DEGs were defined as those with a fold change of ≥2 and a Benjamini-Hochberg–controlled false discovery rate of <0.05. Functional enrichment analysis was performed to identify significant Gene Ontology (GO) terms and using Metascape^84^. Immquant was used to infer alteration in immune cell frequency based on tissue transcriptional profiles^85^.

### Intestinal pathology imaging

Tissue samples from each animal were collected from the mid ileum, and descending colon. The tissues were placed in 10% neutral buffered formalin at room temperature for 48–72 h and processed in a Tissue Tek VIP 5 Tissue Processor. The blocks were paraffin-embedded, cut, and stained with hematoxylin and eosin. Representative images were captured at ×10 magnification by a blinded pathologist.

### 16S amplicon sequencing

Total DNA was extracted from ~0.25 g of luminal contents using the PowerSoil DNA Isolation Kit (MO BIO Laboratories, Carlsbad, CA, USA). Next, we amplified the hypervariable V4-V5 region of the 16S rRNA gene using PCR primers (515 F/926 R with the forward primers including a 12-bp barcode)^86^. PCR reactions contained 12.5 μl GoTaq mastermix, 9.5 μl nuclease-free H_2_0, 1 μl template DNA, and 1 μl 10 μM primer mix. Thermal cycling parameters were 94 °C for 5 min, 35 cycles of 94 °C for 20 s, 50 °C for 20 s, 72 °C for 30 s, followed by 72 °C for 5 min. PCR products were purified using a MinElute 96 UF PCR Purification Kit (Qiagen, Valencia, CA, USA). Libraries were sequenced (2 ×300 bases) using an Illumina MiSeq.

Raw FASTQ 16S rRNA gene amplicon sequences were uploaded and processed using the QIIME2 analysis pipeline^87^. Briefly, sequences were demultiplexed and the quality filtered using DADA2^88^, which filters chimeric sequences and generates an amplicon sequence variant (ASV) table equivalent to an operational taxonomic unit (OTU) table at 100% sequence similarity. Sequence variants were then aligned using MAFFT^89^. and a phylogenetic tree was constructed using FastTree2^90^. Taxonomy was assigned to sequence variants using q2-feature-classifier^91^. against the SILVA database (release 119)^92^. To prevent sequencing depth bias samples were rarified to 13,000 sequences per sample before alpha and beta diversity analysis. QIIME 2 was also used to generate the following alpha diversity metrics: richness (as observed taxonomic units), Shannon evenness, and phylogenetic diversity. Beta diversity was estimated in QIIME 2 using weighted and unweighted UniFrac distances^93^.

### Shotgun metagenomic library preparation and analysis

Shotgun metagenomic libraries were prepared from 50 ng of gDNA using the Nextera library prep (Illumina, La Jolla CA) per Illumina’s recommended protocol and sequenced on an Illumina HiSeq 4000 2 × 100. Raw demultiplexed reads were quality filtered using Trimmomatic^94^, and potential host reads were removed by aligning trimmed reads to the *Macaca mulata* genome (Mmul 8.0.1) using BowTie2^95^. Trimmed and decontaminated reads were then annotated using the HUMAnN2^96^. pipeline using default settings with the UniRef50 database and assigned to Metacyc pathways. Functional annotations were normalized using copies per million (CPM) reads before statistical analysis^97–99^. Species-level taxonomy was assigned to quality-controlled short reads using Metaphlan2^100^. Genome assemblies were generated for each sample. Trimmed and decontaminated reads were assembled into contigs using meta-SPAdes with default parameters^101^. and binned into putative genomes using MetaBat^102^. Genome completeness/contamination was tested using CheckM^39^, and all bins with a completeness >80% and contamination <2% were annotated using PATRIC^103^. Taxonomy of draft genomes was determined using PATRICs’ similar genome finder.

### Statistical analysis

All statistical analyses were conducted using PRISM (V8) or the R package Vegan^104^. Bray Curtis dissimilarity matrices were constructed for both species-level relative abundance and normalized gene annotations using the vegdist function in the R package Vegan. Principal coordinate analysis (PcoA) was conducted using the R function cmdscale. PERMANOVAs were performed using the Vegan function ADONIS. Unpaired *t*-test, 1-way, and 2-way ANOVA were implemented using PRISM8 were noted to generate p-values and utilizing the corresponding post-hoc-test when the initial ANOVA was significant. The LEfSe algorithm was used to identify differentially abundant taxa and pathways between groups with logarithmic Linear discriminant analysis (LDA) score cutoff of 2^99^.

Spearman rank correlations were used to investigate the relationship between Lamina propria immune cell populations and the taxonomy of the luminal contents collected from the same site. The “rcorr” function from the R package Hmisc to calculate Spreaman rho and *p*-values^105^. These values were then plotted using the R package corrplot^106^.

## Supplementary Information

Supplementary Information

Supplementary Table1

Supplementary Table1–7

## Data Availability

The sequencing datasets generated for this study are available in the NCBI SRA repository, under the bioproject ID no. PRJNA729051.
