# Supplementary material for: Growth faltering regardless of chronic diarrhea is associated with mucosal immune dysfunction and microbial dysbiosis in the gut lumen
Source: Mucosal Immunol. Author manuscript; Available in PMC 2021 Dec 22. (PMC8379072; doi:10.1038/s41385-021-00418-2)
Supplement: 1707048_Sup_Fig [file NIHMS1707048-supplement-1707048_Sup_Fig.pdf]

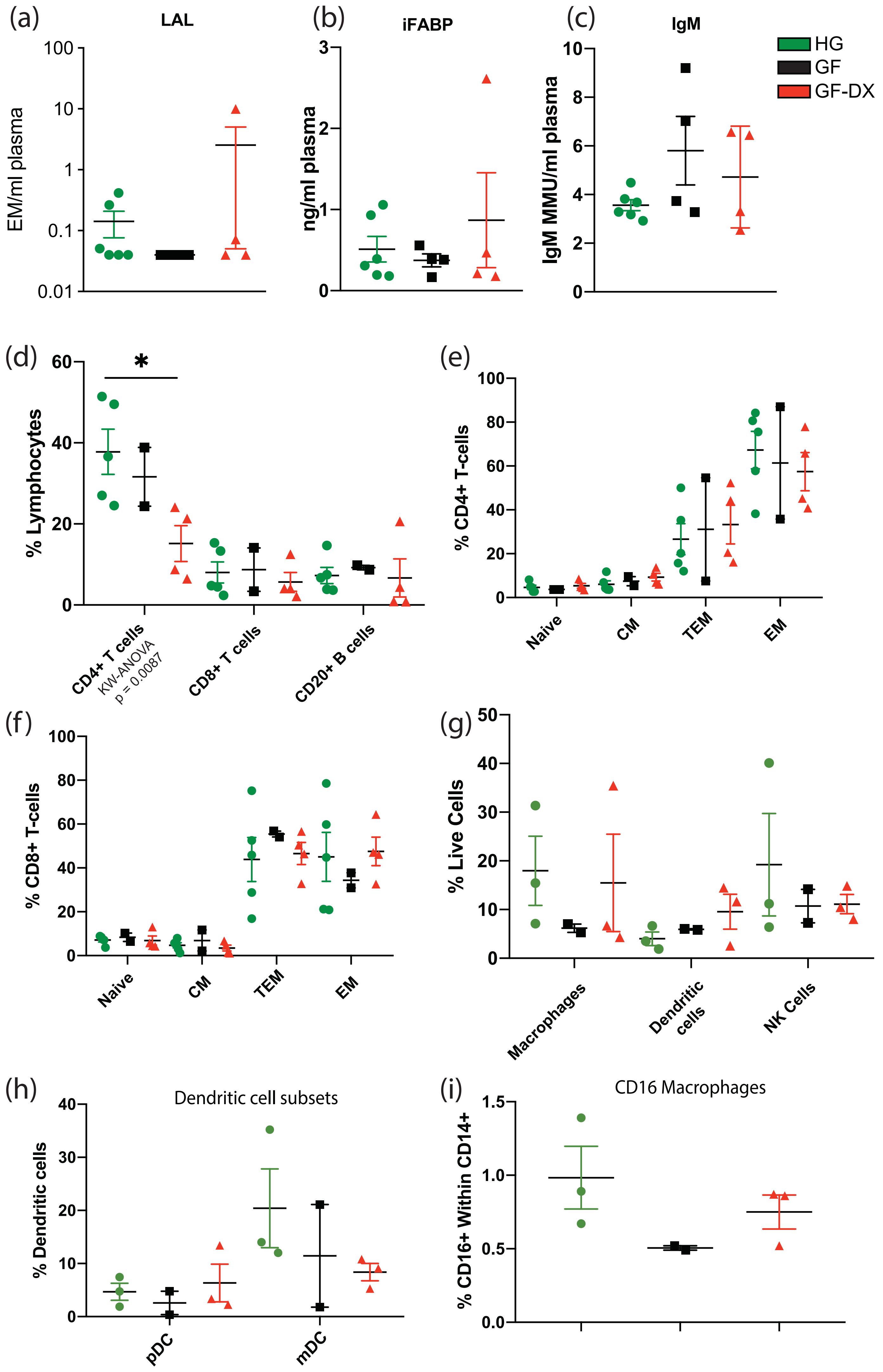

Supplemental Figure 1: Markers of systemic inflammation and ileal immune cell subsets. (a) Limulus amebocyte lysate (LAL), a measurement of circulating free LPS, (b) Fatty acid-binding protein, a marker of barrier dysfunction in the gut, and (c) IgM bound endotoxin in HG, GF, and GF-DX infants. (d) Percent abundance of lamina propria T and B cells in the ileum as measured by flow cytometry. Scatter plots denoting percent abundance of ileal lamina propria (e) CD4+ and (f) CD8+ T-cell naïve and memory subsets, (g) dendritic cells (DC), macrophages, and natural killer (NK), (h) mDCs and pDC subsets, and (i) CD16+ Macrophages. Each point represents a study animal. Horizontal bars and whiskers indicate the mean +/- SEM. Significance was determined using Kruskal Wallis non-parametric ANOVA, with Dunn's multiple comparison * = $p < 0.05$, ** = $p < 0.01$. Post-hoc comparisons were made between HG vs. GF and HG vs. GF-DX.

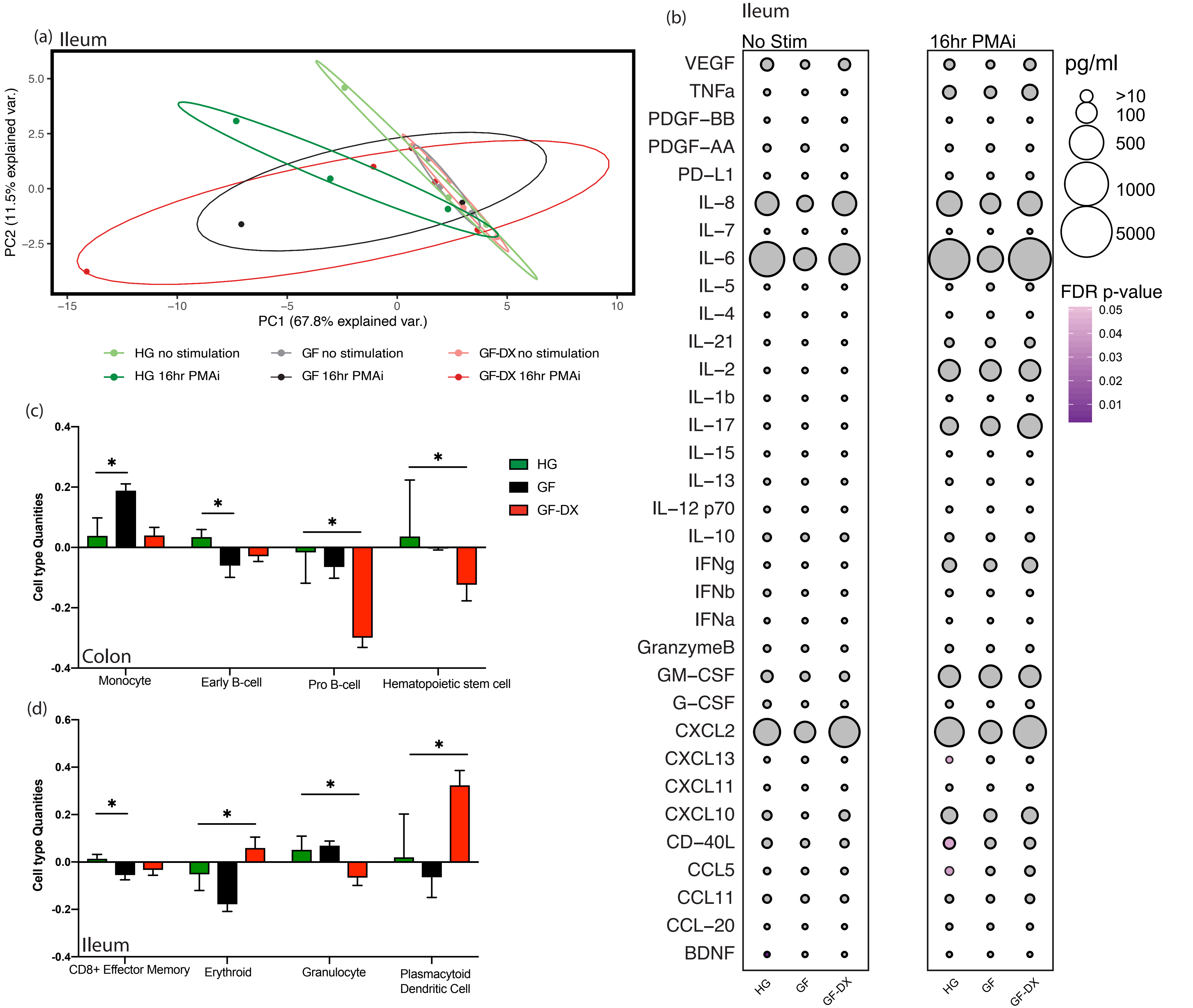

Supplemental Figure 2: Immune mediator production by ileal LPLs. (a) Principal component analysis generated from the levels of 33 immune mediators released by colonic LPLs collected from HG, GF, and GF-DX infants in the absence and presence of PMA/ionomycin. (b) Bubble immune factor production (picograms per milliliter) in the presence or absence of PMA and ionomycin stimulation by colonic LPLs. The size of each circle indicates the mean concentration of the indicated analyte. No analytes were significantly different between the 3-groups with or without PMAi stimulation, as measured by Kruskal Wallis non-parametric ANOVA. immquant was used to predict changes in immune cell populations based on transcriptional profiles of biopsies collected from the (c) colon, and (d) ileum. Significance was determined using 1-way ANOVA with post-hoc Sidak multiple comparisons test * = p <0.05.

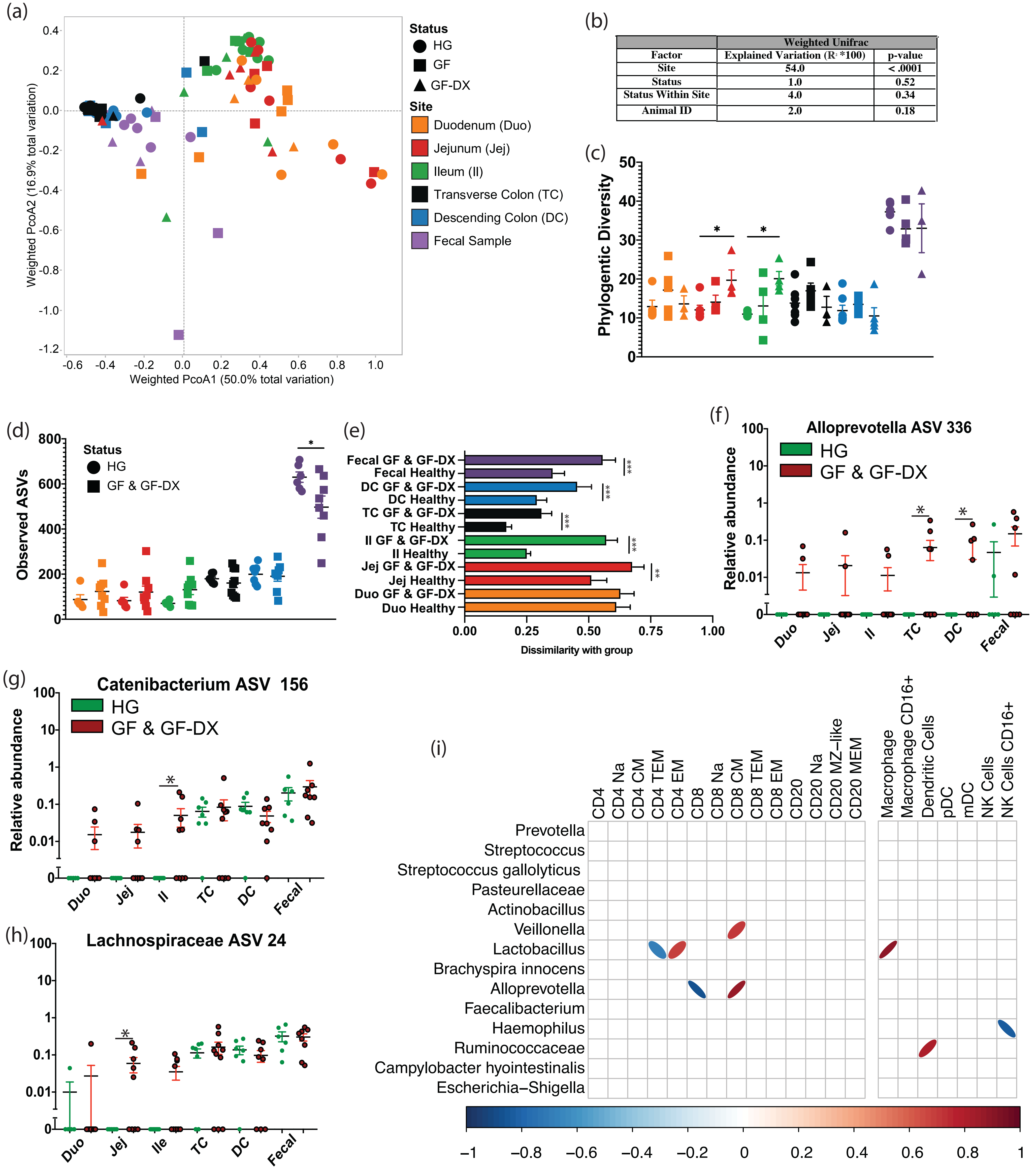

Supplemental Figure 3: Taxonomic landscape of the luminal microbiome and disease related trends. (a) Principal coordinate analysis (PcoA) of weighted UniFrac distances between microbial communities colored by sampling site. (b) The contribution of sample site, host-status, and individual to the total variance in the weighted and unweighted UniFrac dissimilarity matrices measured using PERMANOVA (Adonis with 10000 permutations). (c) Scatter plot of Faiths Phylogenetic Diversity at each sample site/host status; each point represents an individual sample with solid lines indicating the median value, and whiskers the SEM. (d) Scatter plot of observed amplicon sequencing variants (ASVs) at each sample site/host status; each point represents an individual sample with solid lines indicating the median value, and whiskers the SEM. (d) Bar graphs illustrating average weighted UniFrac distances between the luminal microbiome of infants at each gut site. (f-h) Scatter plots of ASVs that displayed differential abundance patterns across gut sites between HG and combined GF & GF-DX infants. Unpaired T-test between HG and combined GF & GF-DX infants at each site, * $p < 0.05$, ** $p < 0.01$, *** $p < 0.001$. (i) Correlation analysis between colonic lamina propria immune cell populations and microbial taxa with > 1% average abundance. Only correlations with uncorrected $p < 0.05$ are shown as ellipses, with blue indicating a negative correlation and red a positive. The width of each ellipse is proportional to the strength of the correlation with narrower ellipse indicating a lower p value.

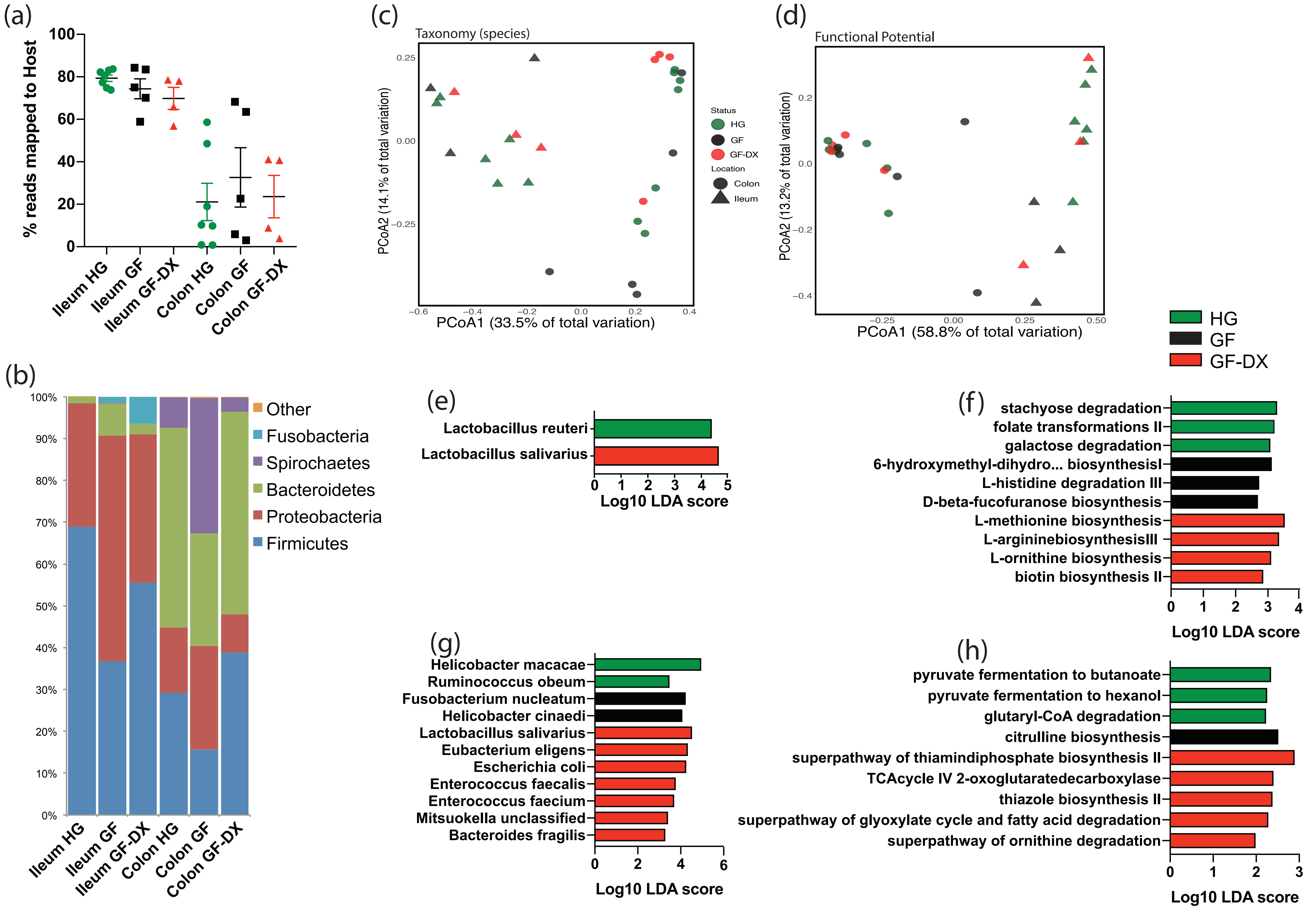

Supplemental Figure 4: The luminal microbiota of infant macaques experiencing growth faltering and/or active chronic diarrhea are taxonomically and functionally distinct. (a) Scatter plot of shotgun metagenomic reads that mapped to the host genome across sampling sites and host status. (b) Phyla plot of luminal contents. All phyla below 1% average abundance grouped into "Other". Bars represent the average for the indicated sampling site. (c) Principal coordinate analysis (PcoA) of Bray-Curtis dissimilarity built on species-level abundance from MetaPhlan2. (d) PcoA of Bray-Curtis dissimilarity built on the abundance of all functional genes annotated using the Uniref50 database. Differentially abundant species (e) and select functional pathways (f) between HG, GF, and GF-DX infants in the ileum. Differentially abundant species (g) and select functional pathways (h) between HG, GF, and GF-DX infants in the colon. Differential abundance was determined using LEFsE (Log10 LDA score > 2).
