# Supplementary material for: Growth faltering regardless of chronic diarrhea is associated with mucosal immune dysfunction and microbial dysbiosis in the gut lumen
Source: Mucosal Immunol. Author manuscript; Available in PMC 2021 Dec 22. (PMC8379072; doi:10.1038/s41385-021-00418-2)
Supplement: 1707048_Sup_info [file NIHMS1707048-supplement-1707048_Sup_info.docx]

**Supplemental Table 1: Cohort info**

**Supplemental Table 2: DEG from ileum and colon- RPKM table for all samples and DEG**

**Supplemental Table 3: 16S gut-site Lefse 3-groups**

**Supplemental Table 4: 16S gut-site Lefse 2-groups**

**Supplemental Table 5: Host mapping stats**

**Supplemental Table 6: Metagenomic Lefse gut-sites**

**Supplemental Table 7: Genomes assembled from luminal contents**
